# OBESE AFRICAN AMERICAN OLDER WOMEN’S EFFECT OF EXERCISE TRAINING ON HEALTH CAPABILITIES AND LIFE SATISFACTION

**DOI:** 10.1093/geroni/igac059.2886

**Published:** 2022-12-20

**Authors:** Dongwook Cho, Sung Kyeom Kim

**Affiliations:** Alcorn State University, Lorman, Mississippi, United States; Dongseo University, Busan, Pusan-jikhalsi, Republic of Korea

## Abstract

The purpose of this study is to determine whether a 12-week exercise course influences obese African American older women’s health capabilities and life satisfaction levels. Participants, who passed an initial screening and pre-assessment, were asked to measure current health capabilities and life satisfaction levels both prior to, and following, the 12-week exercise training. Researchers found that improvement in health capabilities, although there were no significant differences following exercise training participation, while obese African American older women had significantly higher life satisfaction levels. The findings suggest that a 12-week exercise training can be beneficial to enhance obese African American older women’s health capabilities and life satisfaction levels. Professionals in the field of gerontology and exercise should provide the appropriate exercise trainings so that obese African American older women would be able to optimize their life satisfaction levels.

